# Potential Molecular Links Between Triclosan Exposure and Prostate Cancer: An Integrative Computational Analysis

**DOI:** 10.3390/ijms27209143

**Published:** 2026-10-15

**Authors:** Hongyang You, Yunlu Zou, Jiatong Liu, Yinghao Xiao, Jixin Li, Jiahui Xu, Wei Xu

**Affiliations:** School of Pharmaceutical Sciences, Changchun University of Chinese Medicine, Changchun 130117, China

**Keywords:** triclosan, prostate cancer, Mendelian randomization, molecular docking, machine learning

## Abstract

Triclosan (TCS) is a widely used antimicrobial and endocrine-disrupting chemical whose potential relevance to prostate cancer (PCa) biology remains insufficiently understood. We integrated multi-omics and computational analyses to identify candidate targets that overlap between predicted TCS-related molecular targets and PCa-related transcriptomic alterations. Cross-database target mapping, differential expression, and network analyses were combined with machine learning, molecular docking, molecular dynamics simulations, Mendelian randomization, and single-cell transcriptomic profiling. Pathway analyses highlighted oxidative stress and metabolic dysregulation as key processes shared between predicted TCS-related targets and PCa-associated transcriptomic alterations. Machine learning prioritized five candidate genes—FERMT2, F5, PRKCA, ABCC4, and ACOX2—with discriminative value for PCa classification in the analyzed cohorts. Docking and molecular dynamics simulations supported plausible binding compatibility of TCS to ABCC4 and ACOX2. Mendelian randomization suggested that genetically predicted ABCC4 expression may be associated with PCa risk. Single-cell analysis further showed cell-type-specific expression of candidate genes in tumor-enriched epithelial populations. These findings provide an integrative framework for generating mechanistic hypotheses on how TCS-related molecular targets may be associated with PCa biology.

## 1. Introduction

Triclosan (TCS) is one of the most widely used synthetic antimicrobial agents and has long been incorporated into personal care products, household cleaners, medical devices, and textiles [1,2]. Owing to its widespread use, TCS has been ubiquitously detected in environmental media and human biological samples, with biomonitoring studies such as NHANES indicating pervasive chronic exposure [3,4].

TCS is highly lipophilic, with a log K_o_w of approximately 4.8, facilitating its accumulation in lipid-rich tissues and endocrine organs [5]. Increasing evidence suggests that TCS acts as a prototypical endocrine-disrupting chemical (EDC). Epidemiological studies have linked urinary TCS concentrations with altered thyroid hormones, sex steroids, and gonadotropins, while animal studies have demonstrated impaired reproductive development and disruption of the hypothalamic–pituitary–gonadal axis following exposure [6,7,8,9].

Mechanistically, beyond its classical interference with steroidogenesis, hormone receptor signaling, and endocrine feedback regulation [10], TCS has been shown to affect multiple signaling pathways associated with energy metabolism and inflammation. Experimental studies demonstrate that TCS acts as a modulator of peroxisome proliferator-activated receptor gamma (PPARγ), altering PPARγ expression and affecting its downstream mTOR signaling in mouse preadipocytes, thereby disturbing cellular metabolic homeostasis [11]. Concurrently, TCS exposure induces production of reactive oxygen species (ROS), regulates inflammatory responses through the PPARγ/NF-κB axis, and alters the expression of nitric oxide synthase (NOS) system, suggesting its potential role in redox imbalance and neuroinflammation [12]. At the lipid metabolism level, in vivo studies in zebrafish further confirm that TCS exposure induces hepatic lipid accumulation through mechanisms involving upregulation of fatty acid synthase, increased fatty acid uptake, and suppression of β-oxidation, contributing to lipid metabolic dysregulation [13]. Notably, dysregulated PPAR signaling, enhanced oxidative stress, chronic inflammation, and lipid metabolic reprogramming are well-established drivers of prostate cancer initiation and progression [14]. However, despite these established effects of TCS on cancer-relevant pathways, the specific molecular mechanisms and candidate targets linking TCS-related molecular alterations with prostate cancer biology remain largely elusive.

PCa is the most frequently diagnosed malignancy among men in many countries and is strongly influenced by hormonal regulation and endocrine signaling. Emerging evidence suggests that environmental EDCs may contribute to PCa initiation and progression through disruption of androgen and estrogen pathways, epigenetic reprogramming, and chronic inflammatory responses [14,15]. Experimental studies have demonstrated that several EDCs can promote proliferation, migration, and therapy resistance in PCa cells via androgen receptor–dependent and independent mechanisms [16]. However, direct mechanistic evidence linking TCS exposure to PCa development is scarce, and the molecular targets mediating its potential pro-carcinogenic effects remain largely unknown.

Given the widespread human exposure to TCS, its bioaccumulative properties, and its documented endocrine-disrupting activity, elucidating its role in prostate carcinogenesis represents an important unmet need in environmental health research. Traditional toxicological approaches focusing on single targets or pathways may be insufficient to capture the complex, multi-level effects of EDCs [17]. Integrative strategies combining network toxicology, multi-omics analyses, molecular docking, and genetic evidence approaches have emerged as powerful tools to systematically identify key molecular mediators and biological pathways underlying chemical–disease associations [18,19].

In this context, to investigate how predicted TCS-related molecular targets may intersect with PCa-associated molecular alterations, we established an integrative computational framework combining network toxicology, transcriptomic differential expression analysis, machine learning-based feature selection, molecular docking and molecular dynamics simulations, Mendelian randomization, and single-cell transcriptomic analysis. These complementary approaches were used to characterize candidate targets, prioritize candidate genes, evaluate potential protein–ligand interactions, assess genetic evidence, and determine cellular localization. Through this framework, we sought to prioritize candidate genes and biological pathways associated with predicted TCS-related targets and PCa biology, providing a basis for future experimental validation and mechanistic investigation.

## 2. Results

### 2.1. Collection of Predicted TCS-Related Targets

The standard molecular structure of TCS was obtained from the PubChem database. Subsequently, target prediction results from multiple sources, including ChEMBL, STITCH, SwissTargetPrediction, and TargetNet, were integrated to identify protein targets potentially associated with TCS. Finally, the target proteins were uniformly annotated and classified using the standardized nomenclature system of the UniProt database, resulting in 398 predicted TCS-related targets (Supplementary Table S1).

### 2.2. Acquisition and Preprocessing of PCa-Related Datasets

**Figure 1:**
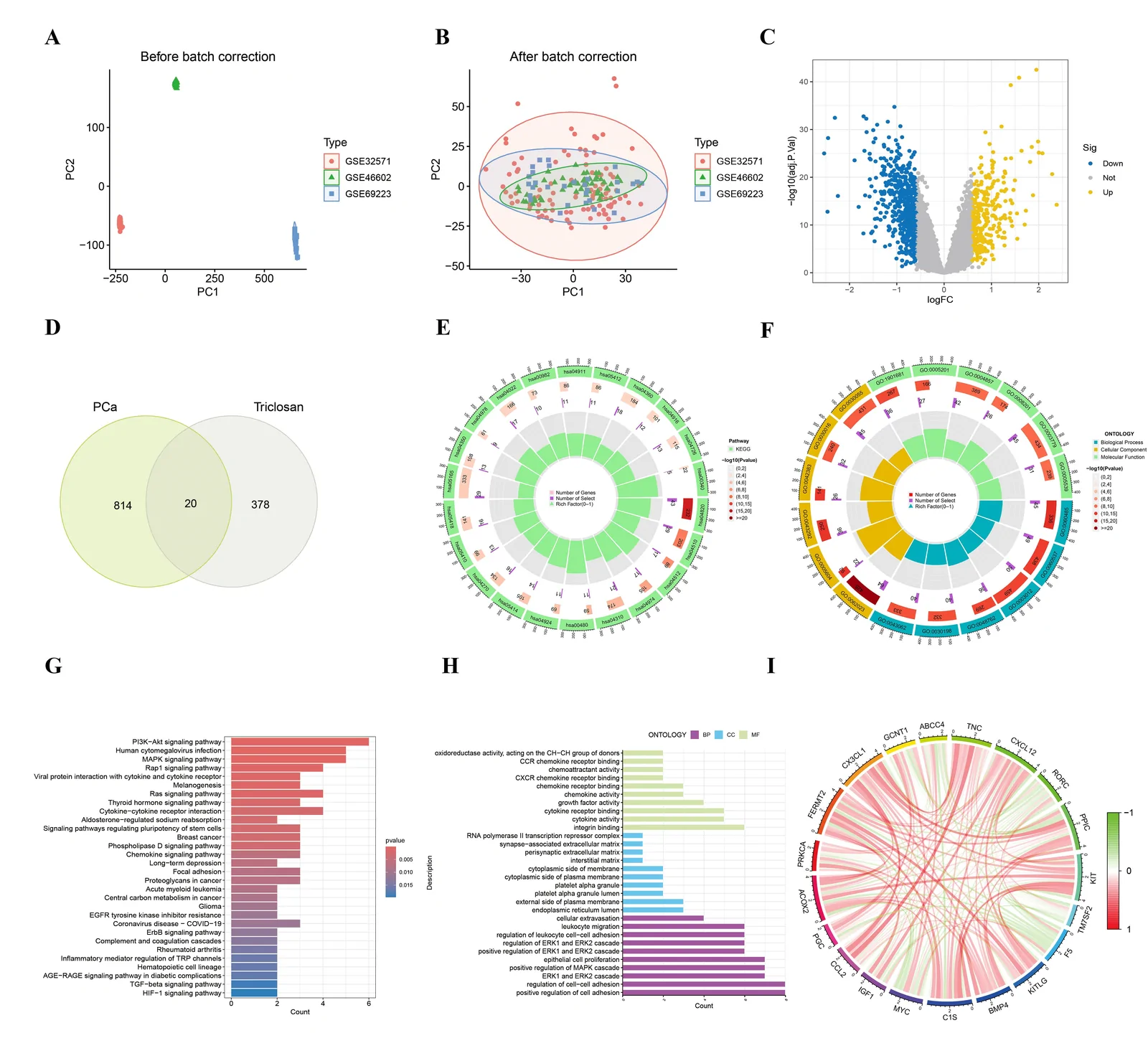
(**A**). PCa plot before batch effect correction of the dataset. (**B**). PCa plot after batch effect correction of the dataset. (**C**). Volcano plot of differentially expressed genes. (**D**). Venn diagram of genes intersecting between TCS and PCa datasets. (**E**). GO enrichment analysis of differentially expressed genes (circular plot). (**F**). KEGG enrichment analysis of differentially expressed genes (circular plot). (**G**). KEGG enrichment analysis of candidate genes (bar plot). (**H**). GO enrichment analysis of candidate genes (bar plot). (**I**). Correlation network of candidate genes.

Three datasets related to PCa—GSE32571, GSE46602, and GSE69223—were screened and obtained from the GEO database (https://www.ncbi.nlm.nih.gov/geo (accessed on 25 November 2025)). These datasets were selected based on their availability of prostate tumor and normal tissue samples and the presence of corresponding clinical annotation information. After preprocessing and normalization, the expression profiles from the three datasets were integrated, and batch effects were corrected using the ComBat function in the sva package, with disease status included as a covariate to preserve biological variation. Principal component analysis (PCA) demonstrated that the dataset-specific distribution was substantially reduced after batch correction, supporting the effectiveness of data integration (Figure 1A,B).

### 2.3. Screening of Differentially Expressed Genes and Enrichment Analysis

The “limma” package was used to filter out differentially expressed genes between the PCa and control groups in the integrated dataset (Figure 1C). A total of 834 genes with significant expression differences were identified by applying strict statistical thresholds (Supplementary Table S2). Enrichment analysis was performed to reveal the molecular function and pathway characterization of the differentially expressed genes (Figure 1E,F). The predicted TCS-related targets were intersected with the genes differentially expressed in PCa datasets, and a Venn diagram was used to identify 20 overlapping candidate genes (Figure 1D), including ABCC4, FERMT2, ACOX2, and others (Supplementary Table S3). KEGG pathway enrichment and GO functional annotation analyses were conducted for this target group (Figure 1G,H).

### 2.4. Chromosomal Localization and Expression Correlation Analysis of Overlapping Candidate Genes

**Figure 2:**
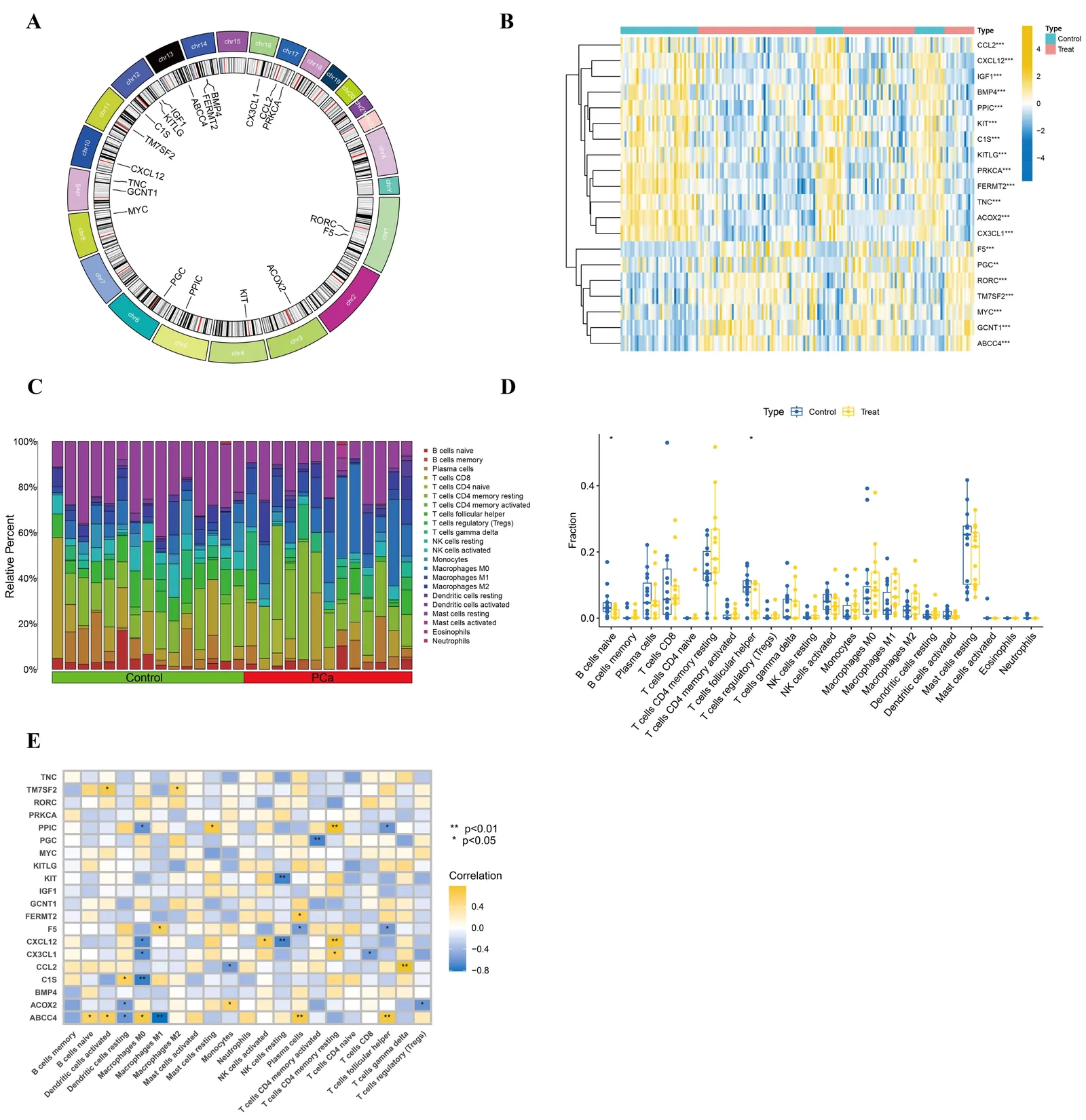
(**A**). Circular plot of chromosomal locations of candidate genes. (**B**). Heatmap of differential expression of candidate genes in normal and PCa samples. ** *p* < 0.01; *** *p* < 0.001. (**C**). Bar plot showing the relative percentage of each immune cell type in the samples. (**D**). Boxplot of immune cell scores in normal and PCa samples. (**E**). Heatmap of correlation analysis between candidate genes and immune cells. * *p* < 0.05; ** *p* < 0.01.

The expression patterns and genomic locations of the 20 overlapping candidate genes were further characterized (Supplementary Table S3). Correlation analysis between pairs of overlapping candidate genes in the PCa samples revealed significant correlations, as shown in Figure 1I. The specific chromosomal locations of the candidate genes are shown in Figure 2A. In the PCa group, ABCC4, PGC, RORC, TM7SF2, MYC, and GCNT1 were highly expressed, while the remaining genes were highly expressed in the normal group (Figure 2B).

### 2.5. Immune Cell Infiltration and Correlation Analysis

Given that functional enrichment analysis of the overlapping candidate genes revealed their involvement in immune-related and inflammatory pathways (Figure 1G,H), we further investigated whether these candidate genes were associated with alterations in the immune microenvironment of PCa. A significantly higher proportion of certain immune cells was observed in the PCa group compared with the normal group. Specifically, resting M2 macrophages and CD4+ memory T cells were highly expressed in the PCa group, while CD8+ T cells showed higher infiltration levels in the normal group, as shown in Figure 2C,D. Immune cell correlation analysis (Figure 2E) indicated that several correlations between candidate genes and immune cells were predominantly positive. Among the immune cells significantly correlated (*p* < 0.05) with the candidate genes, M0 macrophages, resting-state NK cells, regulatory T cells, and CD8+ T cells were mainly negatively correlated with the relevant candidate genes. In contrast, activated dendritic cells, M2 macrophages, gamma delta T cells, resting CD4+ memory T cells, and initial B cells were positively correlated with the relevant candidate genes.

### 2.6. Clustering Analysis of Candidate Genes and Characterization of Molecular Clusters

**Figure 3:**
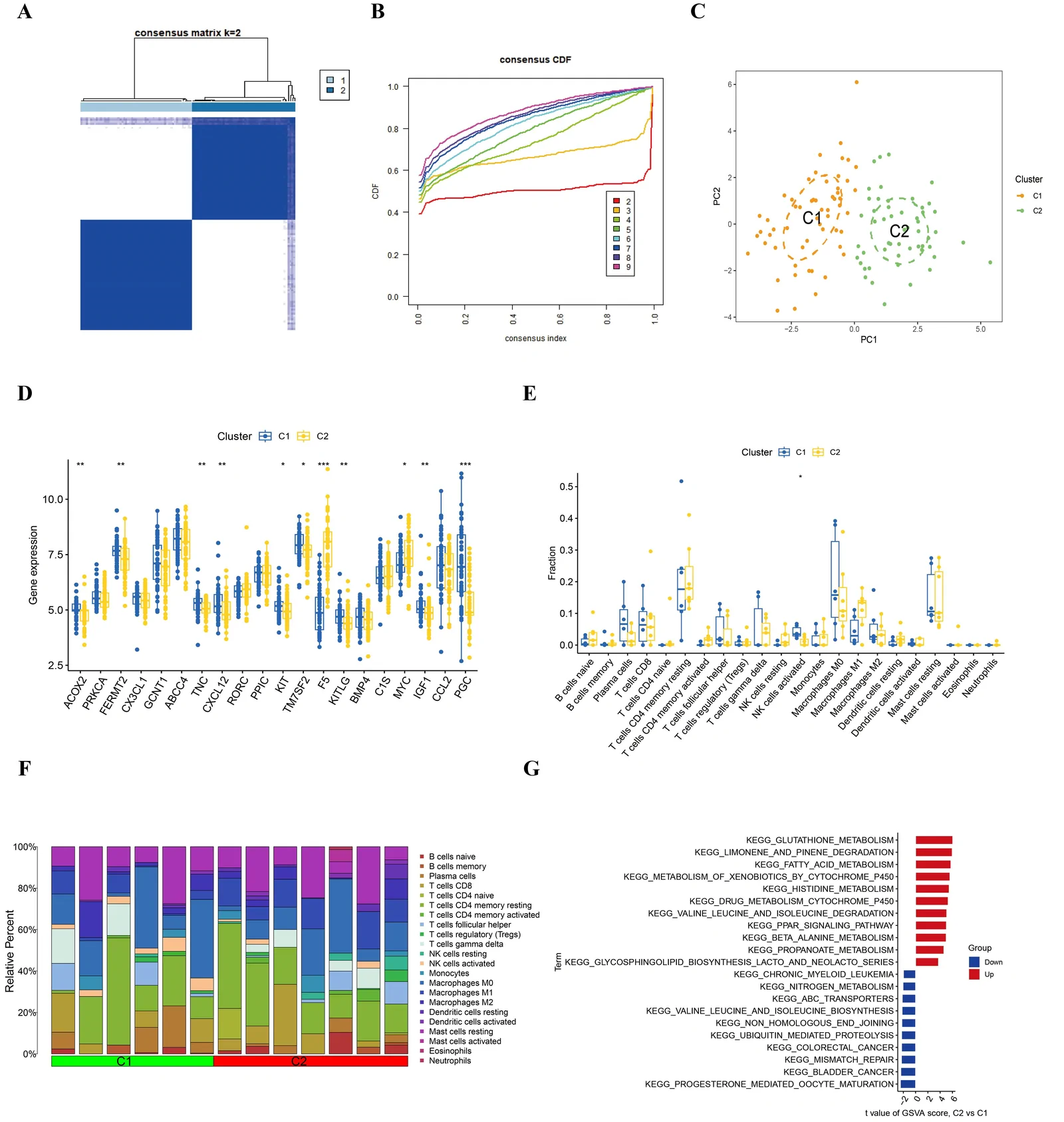
(**A**). Consensus matrix heatmap for candidate gene clustering. (**B**). Consensus cumulative distribution function (CDF) plot for candidate gene clustering. (**C**). PCa scatter plot of expression differences between core gene clusters. (**D**). Boxplot of differential expression analysis between core gene clusters. (**E**). Boxplot of immune cell scores between core gene clusters. (**F**). Bar plot showing the relative percentage of each immune cell type in core gene clusters. (**G**). GSVA GO term bar plot between clusters. * *p* < 0.05; ** *p* < 0.01; *** *p* < 0.001.

Consensus clustering implemented in ConsensusClusterPlus with k-means as the internal clustering algorithm was applied to cluster the candidate genes. Based on the consensus cumulative distribution function (CDF), K = 2 was selected as the optimal number of clusters (Figure 3B). The PCa samples were categorized into two groups, C1 and C2 (Figure 3A). The expression of candidate genes between the two groups was then assessed. The results showed (Figure 3D) that ACOX2, CXCL12, PGC, and FERMT2 exhibited higher expression in C1 compared to C2. PCA (Figure 3C) demonstrated clear separation between C1 and C2 clusters. The CIBERSORT algorithm was used to assess immune cell differences between C1 and C2. The results indicated that macrophages M0 and activated NK cells were upregulated in C1 (Figure 3E,F). GSVA pathway analysis (Figure 3G) showed that fatty acid metabolism was upregulated in C1, while glutathione metabolism, the PPAR signaling pathway, and ABC transporters were mainly upregulated in C2. Nitrogen metabolism, among other pathways, was downregulated in C2 compared to C1.

### 2.7. WGCNA Analysis of Candidate Gene-Associated Modules

**Figure 4:**
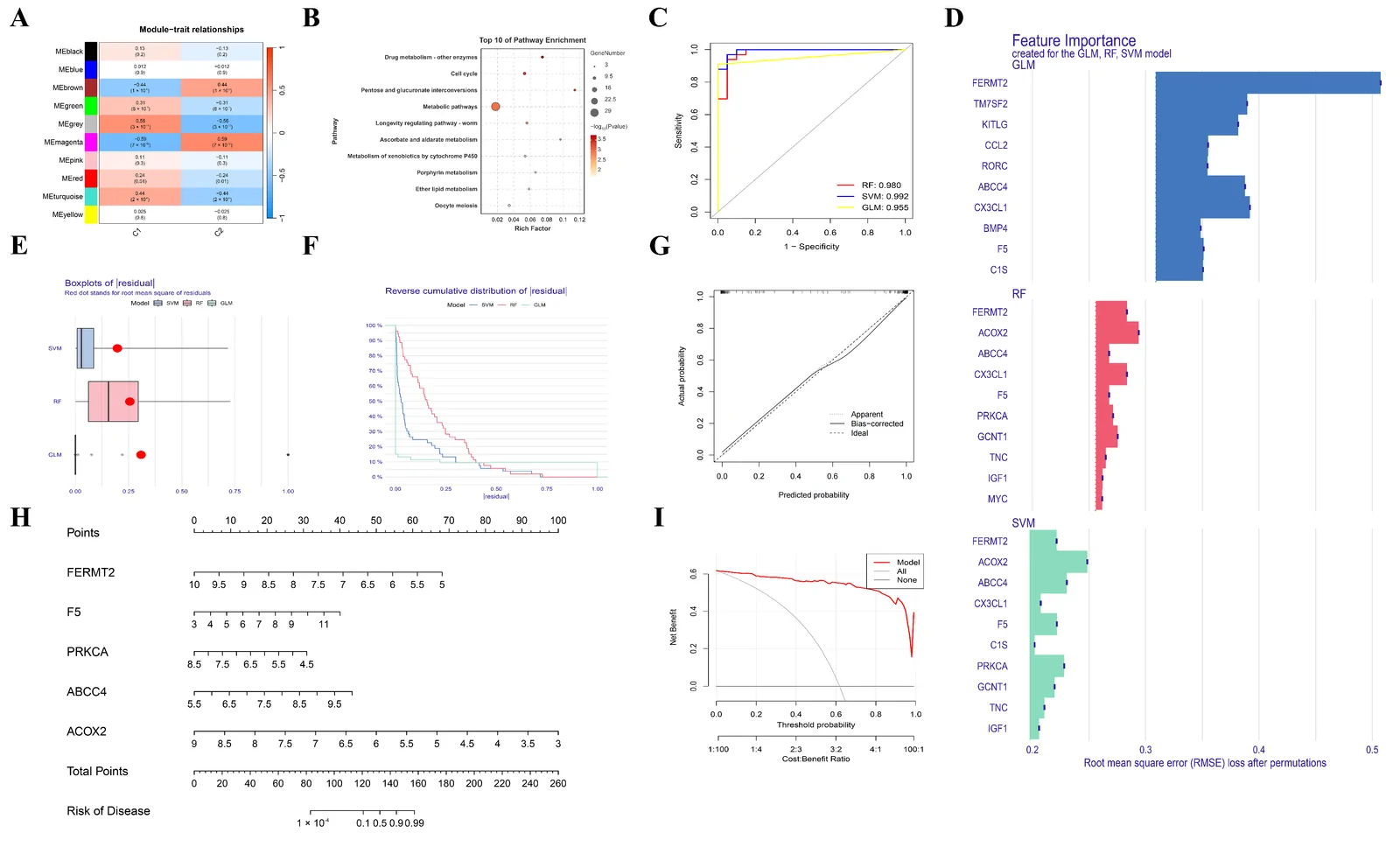
(**A**). Heatmap of the relationship between clustering WGCNA modules and traits. (**B**). Bubble plot of enrichment analysis for core WGCNA modules. (**C**). ROC curve for the three machine learning models. (**D**). Bar plot of feature importance for the three machine learning models. (**E**). Boxplot of residuals for the three machine learning models. Grey dots show individual sample absolute residuals, and red dots denote the residual root mean square error (RMSE) of each model. (**F**). Inverse cumulative distribution of residuals for the three machine learning models. (**G**). Calibration curve for the predictive feature genes in PCa. (**H**). Nomogram for the predictive feature genes. (**I**). Decision curve for the predictive feature gene profile in PCa.

Based on the selected soft threshold of 6, the neighboring values were converted into a topological overlap matrix (TOM) using the topological overlap matrix similarity function with the appropriate power values. The genes were then classified into different co-expression modules. The minimum module size was set to 100, and modules with a correlation < 0.25 were merged using dynamic tree cutting. Ultimately, all genes were divided into several modules. The relationship between modules was assessed by calculating the correlation between modules and traits, and a heat map was drawn (Figure 4A). Among the 10 modules, the magenta module was identified as the key module related to clustering (r = 0.59, *p* = 7 × 10^−12^) and contained 200 genes. Core module enrichment analysis revealed (Figure 4B) that the top ten pathways included Metabolic pathways and Ascorbate and aldarate metabolism, with Metabolic pathways being a core feature of PCa.

### 2.8. Selection of Machine Learning Models and Construction of PCa Classification Models

Three machine learning models (SVM, RF, and GLM) were constructed to classify PCa versus normal samples. The dataset was randomly divided into a 70% development set and a 30% internal test set using stratified sampling (random seed = 1234). Within the development set, repeated 5-fold cross-validation (10 repeats) was applied for model training and parameter optimization, with ROC AUC as the primary evaluation metric. The independent internal test set was held out for final performance assessment. Within this validation setting, the SVM model demonstrated the best overall discriminative performance, showing the highest AUC and more favorable residual distribution patterns compared with RF and GLM (Figure 4C,E,F). Feature-importance analysis based on the SVM model identified FERMT2, F5, PRKCA, ABCC4, and ACOX2 as the top-ranked genes contributing to classification (Figure 4D). An exploratory nomogram based on these five genes was subsequently constructed, and its calibration and decision-curve characteristics were assessed within the analyzed dataset (Figure 4G–I). These analyses were used for internal classification and candidate-gene prioritization rather than for establishing a clinically validated diagnostic model.

### 2.9. TCGA Expression Analysis and Molecular Docking of Candidate Genes

ABCC4 and F5 expression levels were significantly higher in tumor samples than in normal tissues. For independent expression-level validation of the selected candidate genes, the TCGA-PRAD cohort was analyzed. In contrast, FERMT2, PRKCA, and ACOX2 (-) exhibited a clear trend of down-regulation in the tumors (*p* < 0.05) (Supplementary Figure S1A–E). To investigate the potential binding interactions between TCS and the predicted core target genes, molecular docking was performed for FERMT2, F5, PRKCA, ABCC4, and ACOX2 using AutoDock Vina. The results showed that ABCC4 (docking score −7.2 kcal/mol) and ACOX2 (−7.1 kcal/mol) showed relatively favorable docking scores, followed by F5 (−6.0 kcal/mol) and FERMT2 (−5.6 kcal/mol). All target proteins except PRKCA exhibited docking scores below the commonly used cutoff for favorable docking scores (−5.0 kcal/mol) (Supplementary Figure S1F–I). Docking analyses identified ABCC4 and ACOX2 as candidates with relatively favorable predicted docking scores among the evaluated proteins. Based on the docking results, ABCC4 and ACOX2 were selected for subsequent molecular dynamics analysis.

### 2.10. Molecular Dynamics Simulations

**Figure 5:**
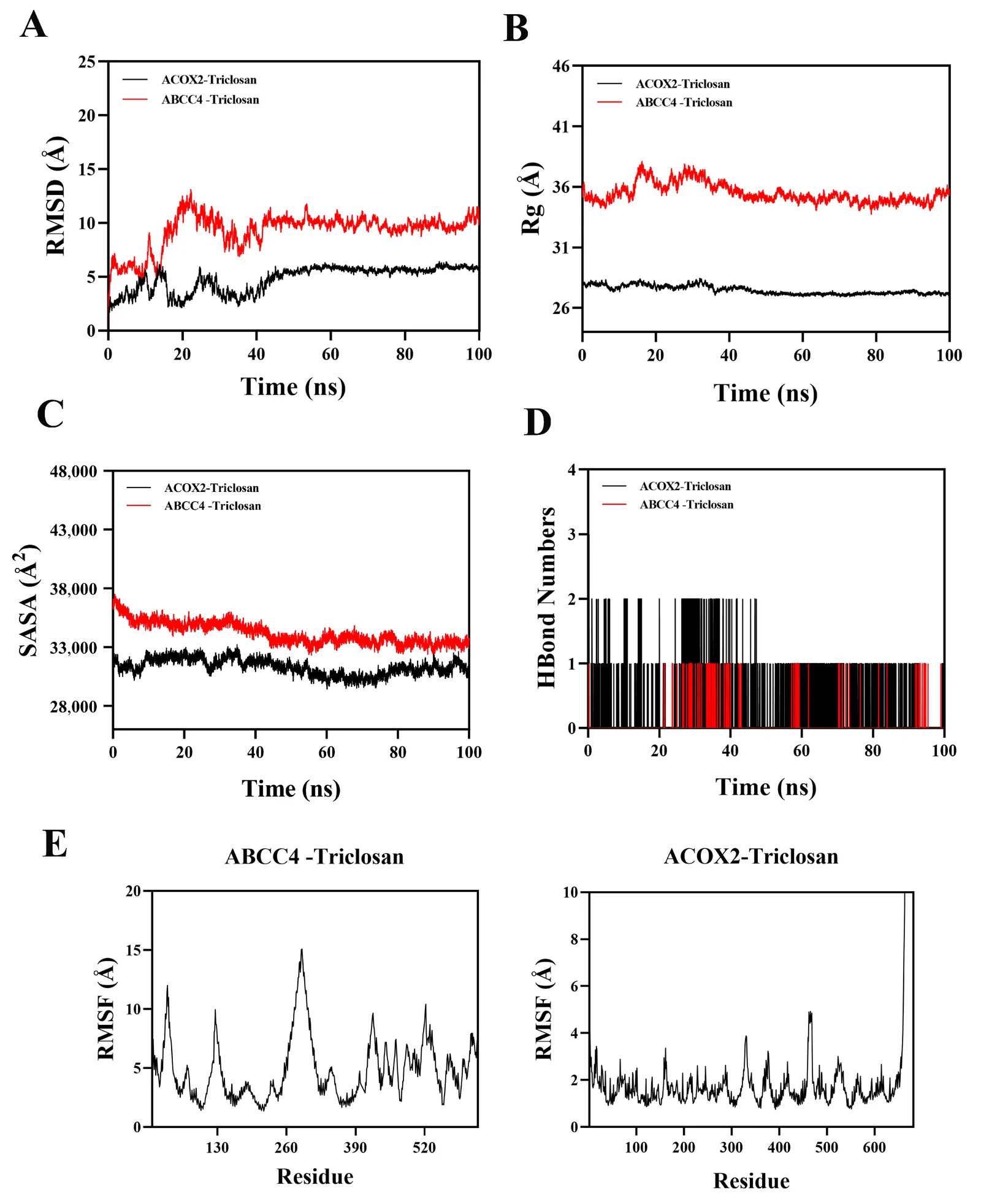
Molecular dynamics simulation analysis of the ABCC4–TCS and ACOX2–TCS complexes. (**A**) Root mean square deviation (RMSD) describing structural deviation and conformational changes over time. (**B**) Radius of gyration (Rg) reflecting the compactness of the protein–ligand complexes during the simulation. (**C**) Solvent accessible surface area (SASA) indicating changes in solvent exposure of the protein–ligand complexes. (**D**) Hydrogen bond interactions between TCS and the target proteins throughout the simulation. (**E**) Root mean square fluctuation (RMSF) showing residue-level flexibility of the ABCC4–TCS (**left**) and ACOX2–TCS (**right**) complexes. All analyses were performed based on 100 ns production MD trajectories. Red lines represent the ABCC4–TCS complex, whereas black lines represent the ACOX2–TCS complex.

To evaluate the conformational dynamics and structural stability of the ABCC4–TCS and ACOX2–TCS complexes, 100 ns molecular dynamics simulations were performed. Both complexes exhibited relatively stable RMSD profiles after the initial fluctuation period, although differences in conformational behavior were observed between the two systems (Figure 5A). The ACOX2–TCS complex maintained a lower RMSD value (~5–6 Å), whereas the ABCC4–TCS complex showed higher fluctuations (~10 Å), suggesting greater conformational flexibility of the ABCC4–TCS complex during the simulation period. RMSF analysis further indicated that residue-level fluctuations were mainly concentrated in specific flexible regions, while most residues exhibited relatively limited fluctuations in both complexes (Figure 5E). The radius of gyration and solvent accessible surface area analyses showed relatively stable trends throughout the simulation, indicating that both protein–ligand complexes maintained relatively stable global compactness and solvent exposure during the simulation period (Figure 5B,C). In addition, both complexes retained protein–ligand hydrogen bond interactions throughout the trajectory, with approximately 1–2 hydrogen bonds observed during most of the simulation time (Figure 5D). Overall, these results suggest that both ABCC4–TCS and ACOX2–TCS complexes maintained relatively stable conformational patterns during the 100 ns simulation period, with ACOX2–TCS displaying lower structural fluctuations and ABCC4–TCS exhibiting greater conformational flexibility.

### 2.11. MR Analysis of Characterized Genes and PCa

**Figure 6:**
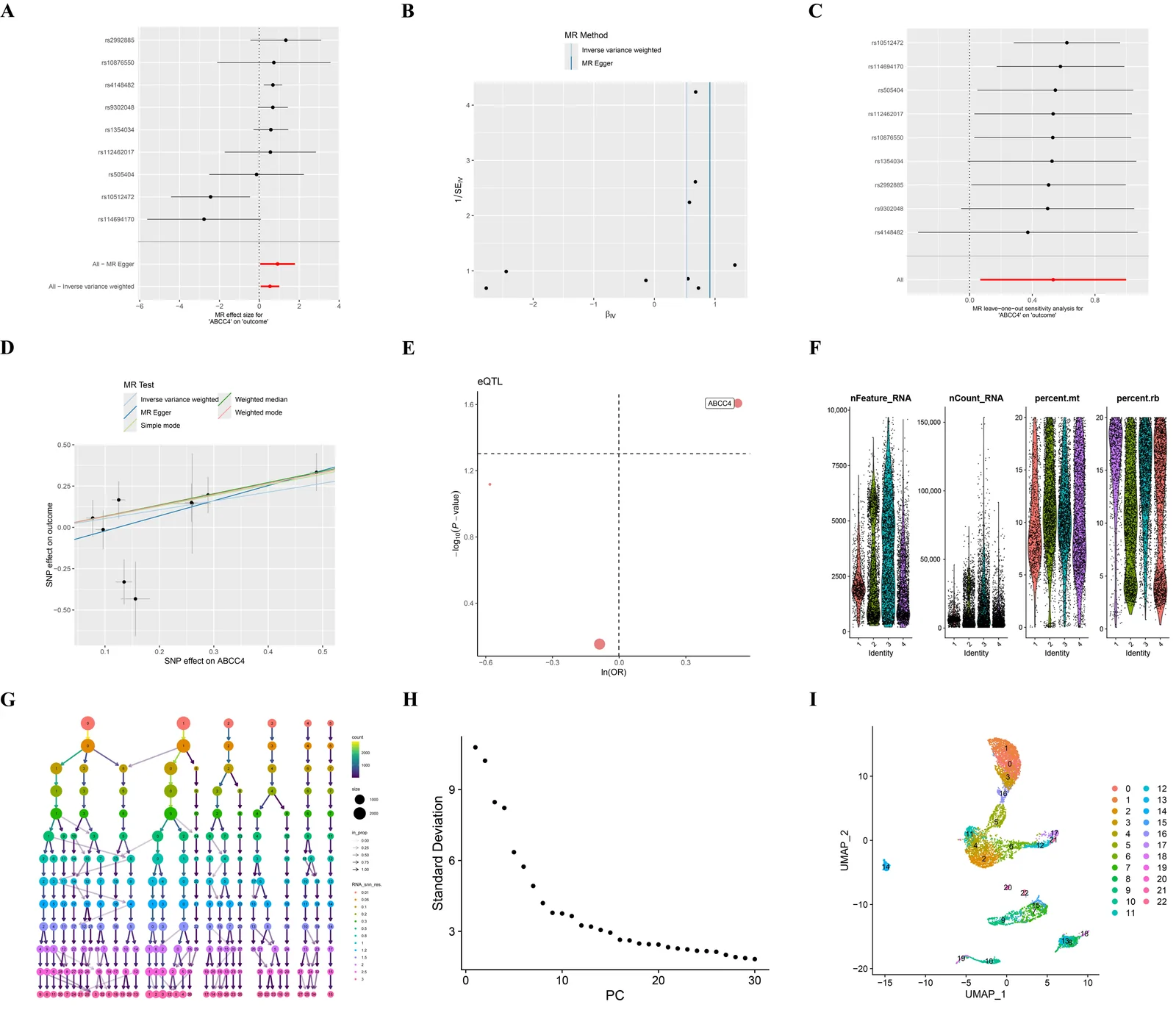
(**A**). Forest plots showing the MR estimates for the association between each SNP in ABCC4 and PCa risk. Black dots and horizontal lines represent the MR effect estimates and their 95% CIs for individual SNPs, respectively. Red dots and horizontal lines represent the overall causal effect estimates and their 95% CIs from MR‑Egger and inverse variance weighted (IVW) analyses, respectively. (**B**). Funnel plot for ABCC4 in PCa. (**C**). Impact of ABCC4 on PCa risk when excluding one SNP at a time. Black dots and horizontal lines represent the MR effect estimates and their 95% CIs for individual SNPs, respectively. Red dots and horizontal lines represent the overall causal effect estimates and their 95% CIs from MR‑Egger and inverse variance weighted (IVW) analyses, respectively. (**D**). Scatter plot of the genetic association between ABCC4 and PCa risk. (**E**). eQTL volcano plot for ABCC4. (**F**). Violin plot of cell identity quality metrics. (**G**). Single-cell clustering dendrogram. (**H**). Elbow plot of PCA. (**I**). UMAP clustering plot of cells without annotation.

Mendelian randomization analysis was performed to evaluate the potential genetic association between genetically predicted candidate-gene expression and PCa risk. Among the four genes with available instrumental variables, genetically predicted higher ABCC4 expression showed a nominal association with increased PCa risk (OR = 1.705; 95% CI, 1.070–2.715; *p* = 0.0247). However, this association did not remain statistically significant after Bonferroni correction for the four tested genes (Bonferroni threshold, *p* < 0.0125). PRKCA, F5, and ACOX2 showed no statistically significant associations with PCa risk (Supplementary Table S4). The IVW analysis for ABCC4 and its association with PCa is shown in Figure 6A,D. Funnel plot analysis revealed that the distribution of SNPs showed a nearly symmetrical pattern (Figure 6B), suggesting the absence of significant heterogeneity, which was further confirmed by Cochran’s Q test (*p* > 0.05) (Supplementary Table S5). The leave-one-out sensitivity analysis showed that the direction of the combined effect of the remaining variants, after excluding individual SNPs one at a time, remained consistent with the overall estimate (Figure 6C), confirming the robustness of the results. In addition, the MR-Egger regression intercept test (*p* = 0.34) did not detect horizontal pleiotropy (Supplementary Table S6), reducing the potential effect of confounders on the association estimate. The eQTL volcano plot for ABCC4 is presented in Figure 6E.

### 2.12. Single-Cell Level Validation of PCa

**Figure 7:**
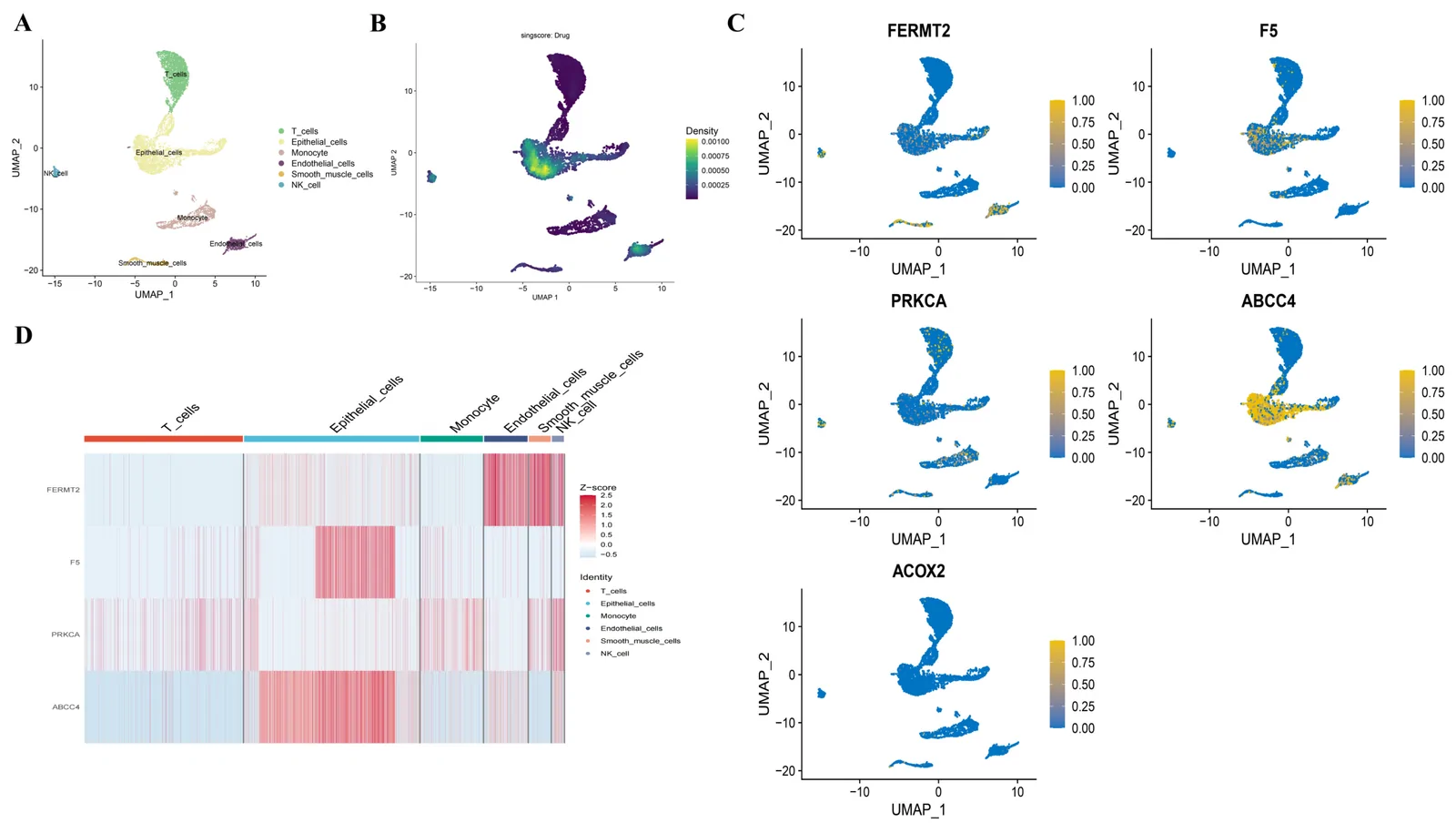
(**A**). UMAP plot with cell type annotation. (**B**). UMAP density plot of predicted TCS-related target gene-set scores. (**C**). UMAP plot of gene expression spatial distribution. (**D**). Heatmap of scaled expression (z-scores) of four detectable candidate genes (FERMT2, F5, PRKCA, and ABCC4) across annotated cell populations.

Single-cell transcriptomic analysis was further performed using the GSE193337 dataset to evaluate the cellular distribution of the selected candidate genes. After quality control and batch correction using the Harmony algorithm, 2000 high-quality cells were retained for downstream analysis. UMAP dimensionality reduction identified distinct cellular populations, which were annotated using the SingleR package and classified into T cells, epithelial cells, monocytes, endothelial cells, smooth muscle cells, and natural killer (NK) cells (Figure 7A). The expression patterns of the selected candidate genes were subsequently visualized across different cell populations. FERMT2, F5, PRKCA, ABCC4, and ACOX2 exhibited distinct distribution patterns among cellular populations, with relatively higher expression observed in specific cell subsets (Figure 7C). To summarize the combined expression of these five genes, a predicted TCS-related target gene-set score was calculated using UCell based on FERMT2, F5, PRKCA, ABCC4, and ACOX2, and this score was relatively enriched in epithelial cells (Figure 7B). This score reflects the relative enrichment of the five-gene signature and does not represent measured cellular TCS exposure. In addition, a heatmap of selected candidate genes across cell populations is shown in Figure 7D. Among the five candidate genes, ACOX2 was not included because its expression was insufficiently detected in this single-cell dataset. This limitation reflects the reduced detectability of low-abundance transcripts in single-cell RNA sequencing, whereas ACOX2 expression was detectable in bulk transcriptomic data.

## 3. Discussion

PCa ranks among the most prevalent malignancies in men worldwide and remains a leading cause of cancer-associated morbidity and mortality [20,21]. Its clinical course ranges from indolent localized tumors to aggressive metastatic disease characterized by skeletal involvement, systemic symptoms, and treatment resistance [22].A central biological hallmark of PCa is its dependence on androgen signaling during both initiation and progression: approximately 90–95% of circulating androgens are of testicular origin and exert their effects primarily through androgen receptor (AR) activation in prostate epithelial cells [23,24]. This mechanistic dependence underpins the clinical effectiveness of androgen-deprivation therapy (ADT) in advanced PCa; however, acquired resistance is common and typically inevitable over the disease course [25,26]. Although prostate-specific antigen (PSA) remains the primary biomarker for screening and monitoring, concerns regarding overdiagnosis and overtreatment highlight the need for improved risk stratification incorporating molecular and environmental factors [27]. In this context, endocrine-disrupting chemicals (EDCs), which can perturb hormone-regulated pathways implicated in prostate carcinogenesis, have received increasing attention [28,29,30].

TCS, a synthetic antimicrobial historically used in personal care products, household items, and certain medical devices, remains widely detectable in biomonitoring studies worldwide despite regional regulatory restrictions [31,32,33]. Data from the U.S. National Health and Nutrition Examination Survey (NHANES) further support population-scale exposure, with urinary TCS concentrations typically spanning low to tens of micrograms per liter—patterns more consistent with chronic, widespread exposure than acute toxicological events. Mechanistically, experimental studies suggest that TCS can interfere with endocrine signaling, oxidative stress responses, and lipid metabolism. In vitro and in vivo evidence suggests that TCS exposure can induce reactive oxygen species (ROS) production, activate NF-κB-mediated inflammatory signaling, and modulate PPARγ activity, a master regulator of lipid metabolism and inflammation [11,12]. In parallel, TCS disrupts fatty acid oxidation and promotes lipid accumulation in multiple models, supporting its potential role in metabolic reprogramming [13]. Additionally, in vitro and animal evidence indicates suppression of testosterone biosynthesis via disruption of steroidogenic gene expression and activation of stress pathways, including autophagy-related mechanisms [34,35,36]. Epidemiologic analyses also report associations between TCS exposure and altered sex hormone profiles in humans, supporting the plausibility of endocrine-disrupting effects in real-world settings [37]. Nevertheless, an important caveat is dose relevance: many mechanistic studies employ micromolar concentrations that likely exceed environmentally observed internal exposure levels, a limitation widely emphasized in low-dose EDC toxicology discussions [38]. Accordingly, our computational findings should be interpreted as hypothesis-generating rather than definitive evidence of in vivo target engagement under environmentally realistic exposure conditions.

Using an integrative framework combining toxicogenomic target prediction, transcriptomic profiling, machine learning, and MR, we identified a set of candidate genes at the intersection of predicted TCS-related targets and PCa-associated transcriptomic alterations [38,39]. Across analytical layers, five candidate genes—ABCC4, FERMT2, F5, PRKCA, and ACOX2—were consistently prioritized, collectively mapping onto major oncogenic processes including xenobiotic transport, metabolic reprogramming, extracellular matrix interaction, and signal transduction. Notably, there is substantial heterogeneity in the strength of supporting evidence across this network: MR suggested a nominal association between genetically predicted ABCC4 expression and PCa risk, although this association did not remain statistically significant after correction for multiple testing [40], whereas links between TCS and ABCC4 or ACOX2 arise primarily from computational target prioritization and structural analyses. Distinguishing these evidence layers is critical for robust interpretation. ABCC4, an ATP-binding cassette transporter involved in drug efflux and broader signaling-relevant transport functions, is particularly compelling given its reported associations with therapy response and cancer progression [41,42]. These properties, together with our integrated results, suggest ABCC4-centered pathways as plausible contributors of environmental exposure–related perturbations in PCa biology, warranting targeted experimental validation. In parallel, FERMT2 and ACOX2 signals further support convergence on metabolic remodeling: FERMT2 downregulation aligns with lipid metabolic reprogramming characteristic of advanced/castration-resistant PCa, while ACOX2—linked to peroxisomal fatty-acid oxidation and bile-acid metabolism—reinforces the metabolic axis implicated by predicted TCS-related targets.

To evaluate structural plausibility, we performed molecular docking and MD simulations assessing potential interactions of TCS with ABCC4 and ACOX2. These analyses suggest that TCS can occupy energetically favorable pockets and form transient stabilizing contacts under dynamic conditions; however, such results demonstrate binding compatibility rather than functional interaction in vivo. Elevated RMSD values may reflect intrinsic conformational flexibility of the protein–ligand complexes during simulation. The relatively stable radius of gyration, solvent-accessible surface area, and hydrogen-bonding interactions suggest that both complexes maintained overall structural stability during the simulated period. However, it should be emphasized that these MD simulations were intended to evaluate the dynamic stability of the docked complexes under the applied computational conditions, rather than to estimate binding affinity, predict physiological binding mechanisms, or infer biological functions. Although the complexes maintained relatively stable conformations during the simulation, these findings should be interpreted only as evidence that the docked poses remained structurally compatible. They do not demonstrate that TCS binds these proteins under physiological conditions or that such interactions produce functional biological effects. Therefore, longer simulations together with experimental validation are required to determine their biological relevance [43].

Immune deconvolution analyses further suggested differences in functional immune subpopulations between PCa cohorts despite relatively modest changes in overall immune infiltration. These transcriptome-derived observations are associative and are best interpreted as exploratory signals reflecting tumor biology–driven shifts in immune composition or functional state rather than direct consequences of TCS exposure. Notably, reduced resting NK cell signatures and increased regulatory T-cell markers correlated with ABCC4 expression patterns, raising the possibility that xenobiotic transport/metabolic programs may intersect with immune modulation within the tumor microenvironment [44]. These findings require validation using orthogonal approaches such as flow cytometry or spatially resolved profiling.

Collectively, our results underscore the potential public health relevance of ubiquitous environmental exposures that may perturb PCa-relevant pathways. Given the high costs and clinical complexity of advanced PCa care, even modest exposure-associated contributions to disease initiation or progression could translate into meaningful population-level impacts. More broadly, this work highlights the value of integrating molecular epidemiology, toxicogenomics, and genetic inference to inform risk assessment and regulatory decision-making.

Despite its integrative design, several limitations warrant emphasis. Reliance on public datasets introduces potential batch effects and cohort heterogeneity, although cross-validation and correction strategies were implemented. While the machine learning models demonstrated robust internal predictive performance, external validation in independent and prospectively collected cohorts is essential to establish generalizability. In addition, consensus clustering was not formally benchmarked against alternative clustering algorithms, and the resulting molecular subgroups should therefore be regarded as exploratory. Critically, although MR suggested a nominal association between genetically predicted ABCC4 expression and PCa risk, it does not establish TCS as the upstream driver of this effect. Likewise, docking and MD simulations provide structural plausibility but do not confirm functional interaction. Future work should prioritize environmentally relevant dose–response experiments, internal dosimetry modeling, and targeted functional assays in prostate-relevant cellular and animal models to test these computational hypotheses. In addition, an explicit lipid bilayer was not included in the ABCC4 MD simulation, the ACOX2 structure was generated by AlphaFold rather than an experimental structure, malignant cell identity was not confirmed by inferCNV, and no wet-lab experimental validation was performed.

## 4. Materials and Methods

### 4.1. Acquisition of TCS Targets

The standard chemical structure of the compound and its SMILES characterization were obtained through the PubChem database using “Triclosan” as the search term [45]. Subsequently, target prediction was carried out based on the ChEMBL database, and the species screening condition was set to “Homo sapiens” to obtain preliminary information on potential targets [46]. To improve target coverage, the obtained SMILES data were imported into both the STITCH database and the SwissTargetPrediction database for complementary searches, and the structural accuracy of the data from different sources was ensured by manual verification and cross-validation of the search results. A standardized processing procedure was implemented for the target information from the above sources: data integration was achieved by matching ChEMBL numbers with STITCH identifiers, and target proteins were uniformly named using the standardized naming system of the UniProt database [47]. A comprehensive database containing information about the secondary targets was eventually created.

### 4.2. Collection of GEO Datasets

Three PCa-related gene expression datasets (GSE32571, GSE46602, and GSE69223) were obtained from the GEO database. The analysis started from probe-level expression matrices (probe × sample) downloaded from GEO rather than raw microarray files (e.g., CEL or IDAT). Probe identifiers were mapped to gene symbols using the corresponding GEO platform annotation files (GPL). Probes lacking valid gene annotations were excluded. When multiple gene symbols were assigned to a single probe, the first annotated gene symbol was retained. For genes represented by multiple probes, probe expression values were averaged at the gene level. Only genes shared among all three datasets were retained for downstream analyses to ensure cross-platform comparability. Each dataset was independently normalized using log2(x + 1) transformation when required, followed by between-array normalization using the normalizeBetweenArrays() function implemented in the limma package(3.58.1) (quantile normalization). The normalized datasets were subsequently integrated, and dataset-associated batch effects were corrected using the ComBat function in the sva package(3.50.0) [48]. Disease status (tumor vs. normal) was included as a covariate in the model matrix to preserve biological variation between tumor and normal samples. Principal component analysis (PCA) was performed before and after batch correction to evaluate changes in dataset-specific variation and residual batch effects [49,50,51]. The selection of these datasets was based on the fact that they contained both prostate tumor and normal prostate tissue samples, had complete processed expression matrices with corresponding platform annotation files, and provided analyzable sample sizes for integrated transcriptomic analysis. Although ComBat substantially reduced dataset-associated batch effects, residual heterogeneity arising from differences in microarray platforms, patient populations, and study-specific protocols cannot be completely excluded. Therefore, the transcriptomic findings should be regarded as exploratory and require validation in independent cohorts.

### 4.3. Differential Gene Screening and Enrichment

Differential gene expression analysis was performed on the merged and batch effect-eliminated datasets using the “limma” package, with differential gene screening thresholds set to (adjusted *p*-value < 0.05 and |log2(Fold Change)| > 1) [52]. Volcano plots were generated to visualize differentially expressed genes between prostate cancer and normal prostate tissue groups. The predicted TCS targets were intersected with the differentially expressed genes in the merged dataset, and a Venn diagram of the intersected genes was generated using code [53]. Gene ontology (GO) and Kyoto Encyclopedia of Genes and Genomes (KEGG) enrichment analyses of differentially expressed genes were performed using the R packages “clusterProfiler(4.10.1),” “enrichmentPlot(1.22.0),” “patchwork(1.2.0),” “GSEABase(1.64.0),” and “GLBase(v1.0).” KEGG signaling pathway enrichment analysis was used to identify pathways significantly associated with genes [54]. Statistical differences were considered significant if the *p*-value was less than 0.05.

### 4.4. Chromosome Location and Expression Correlation Analysis

The core gene expression profiles of the normal and PCa groups were extracted. After statistical screening (*p* < 0.05), significantly differentially expressed candidate genes were identified. Box plots of paired samples were generated using the R package “ggpubr(0.6.0),” and a heat map of differential gene clustering was constructed using the “pheatmap(1.0.12)” package, Perl scripts were used to resolve the physical coordinates of their chromosomes, and a circular genome distribution map was constructed using the “RCircos(1.2.2)” package to demonstrate the localization features [53]. The Pearson correlation coefficient matrix between the candidate genes was further calculated using the “cor” function, and the co-expression regulatory network was visualized with heatmap gradient coloring.

### 4.5. Infiltration, Differences, and Correlation of Immune Cells in PCa Samples

The relative immune cell content of the samples was analyzed by back-convolution of the samples using the CIBERSORT algorithm [55], combined with a 1000 permutation test to improve the confidence of the results, followed by stacked bar graphs to show the distribution of the immune cell composition of each sample. The ssGSEA algorithm was used to quantify the level of immune cell infiltration in individual samples, and single-sample gene set enrichment analysis (ssGSEA) was performed using the R packages “GSVA(1.50.5)” and “GSVABase(1.64.0).” Differences in immune cell content between the normal and PCa groups were compared using the Wilcoxon rank-sum test, and box plots were used to display immune cell subtypes with significant intergroup differences. *p*-values from immune-cell comparisons/correlations were adjusted using Benjamini–Hochberg FDR. Finally, Spearman correlation analysis was used to explore the association between core gene expression and ssGSEA scores based on heatmap visualization of the correlation coefficient matrix [56].

### 4.6. Clustering of Candidate Genes and Inter-Cluster Differential Analysis

Consensus clustering was performed using the ConsensusClusterPlus package(1.66.0) based on the expression profiles of differentially expressed candidate genes from tumor samples only. The maximum number of clusters was set to 9 (K = 9), using the k-means algorithm and Euclidean distance metric (reps = 50, pItem = 0.8, pFeature = 1, clusterAlg = “km”, distance = “Euclidean”, seed = 123,456). A consensus matrix heatmap was generated to visualize clustering stability and sample assignment, whereas box plots were used to compare candidate-gene expression between clusters. K = 2 was selected based on the consensus cumulative distribution function (CDF) and overall cluster stability. Principal component analysis (PCA) was performed to visualize sample separation between molecular clusters [57]. ssGSEA was performed to quantify relative immune-cell infiltration across the identified molecular subgroups, and significant differences were assessed using the Wilcoxon rank-sum test. Finally, KEGG gene sets were obtained from the Molecular Signatures Database (MSigDB), and the GSVA package(1.64.0) was used to calculate gene-set variation scores, with subtype-specific pathway activities visualized using hierarchical clustering heatmaps [58]. Because the identified molecular subgroups were derived solely from transcriptomic clustering, they should be regarded as exploratory. A formal comparison with alternative clustering algorithms was not performed, and independent validation is therefore required.

### 4.7. WGCNA Clustering Analysis of Candidate Genes

The expression matrix was initially subjected to strict quality control to eliminate low-expression genes and abnormal samples. After normalization, Pearson correlations were calculated for all gene pairs. We then selected the soft-thresholding power β based on the scale-free topology criterion (R^2^ ≥ 0.8), aiming for a value where the R^2^ approached 0.9 while still retaining moderate mean connectivity. The chosen β was used to construct the weighted adjacency matrix, which was then transformed into a topological overlap matrix (TOM) for downstream network analysis.

In the module delineation and screening stage, hierarchical clustering was performed based on the topological overlap matrix (TOM), and the dynamic tree cutting algorithm was used to adaptively delineate gene modules. Modules with highly similar feature gene correlations (≥0.75) were merged and color-coded, with gray modules representing unclassified genes. Key modules were identified through Pearson correlation analysis of module characteristic genes (ME, the first principal component) with phenotypes, using a screening criterion of r > 0.5 and an FDR-corrected *p*-value < 0.05.

At the functional resolution stage, GO/KEGG enrichment analysis was performed on the core modules. Significant pathways were identified through a hypergeometric test (*p* < 0.05, Q < 0.1), and cross-module correlation heatmaps were created to reveal functional module interactions [59].

### 4.8. Machine Learning Classification and Exploratory Nomogram Construction

To distinguish PCa samples from normal controls and identify key signature genes with discriminative ability, three machine learning classification models, including random forest (RF), support vector machine (SVM), and generalized linear model (GLM), were constructed based on core gene expression profiles. The dataset was randomly divided into a 70% development set and a 30% internal test set using stratified sampling (random seed = 1234). Within the development set, repeated 5-fold cross-validation (10 repeats) was applied for model training and parameter optimization, with ROC AUC as the primary evaluation metric. The independent internal test set was used for final performance assessment. The objective of machine learning analysis was to evaluate the classification capability of candidate genes and identify key genes through feature importance analysis rather than to establish a clinical diagnostic model. Model performance was comprehensively assessed using ROC curves, AUC values, and residual distribution analyses. The optimal-performing model was selected, and a predictive nomogram integrating the identified key biomarkers was subsequently constructed [60].

### 4.9. TCGA Expression Validation and Molecular Docking

For independent expression-level validation of the selected candidate genes, gene-expression data from the TCGA-PRAD cohort were analyzed. Expression profiles of the identified key genes were extracted from the normalized TCGA-PRAD matrix, and additional log2 transformation was performed when required. Differential expression between tumor and normal tissues was assessed using the Wilcoxon rank-sum test and visualized using the ggplot2(3.5.1) package. For molecular interaction analysis, ligand information was obtained from PubChem, and receptor structures were obtained from the Protein Data Bank (PDB), AlphaFold Protein Structure Database, and UniProt. Molecular docking was performed using AutoDock Vina(1.2.5), and docking scores were interpreted as comparative indicators of predicted ligand–protein interaction strength rather than experimental binding affinity or binding free energy [61,62]. The human ABCC4 structure (PDB ID: 8XOK, resolution: 2.84 Å) was prepared by removing non-protein molecules, adding hydrogens, correcting bond orders, optimizing protonation states, and energy minimization. The docking cavity was defined based on reported substrate-binding regions and pocket prediction analysis, without explicit lipid membrane construction during docking. For ACOX2, the AlphaFold-predicted structure (AF-Q99424-F1, global pLDDT = 94.41) was used because no suitable experimental structure was available, and the binding region was determined according to pocket prediction and known functional regions of the ACOX family. The top-ranked docking poses without apparent steric clashes were selected for subsequent molecular dynamics simulations.

### 4.10. Molecular Dynamics Simulations

To evaluate the dynamic stability and conformational behavior of the docked ABCC4–TCS and ACOX2–TCS complexes, 100 ns molecular dynamics (MD) simulations were performed separately for each complex using GROMACS 2022 [63]. Protein and ligand parameters were prepared using the AMBER14SB and GAFF2 force fields, respectively [64]. Each complex was solvated in a 1 nm TIP3P water box and neutralized with counterions. For the ABCC4 system, an explicit lipid bilayer was not included in the current simulation setup. Following energy minimization and equilibration, 100 ns production simulations were performed under NPT conditions at 310 K. Structural stability and dynamic properties were evaluated using backbone RMSD, RMSF, radius of gyration (Rg), solvent-accessible surface area (SASA), and hydrogen-bond analysis. The current MD simulations were intended to evaluate the dynamic stability of the docked complexes under the applied simulation conditions, rather than to estimate binding affinity, predict physiological binding, or infer biological function. Therefore, within the limitations of the present setup, the MD analyses should be interpreted as providing supportive evidence for the structural compatibility of the docked poses. They do not confirm physiological binding mechanisms or substitute for experimental validation.

### 4.11. Mendelian Randomization Analysis of Candidate Genes Associated with PCa Risk

To evaluate the genetic association between candidate gene expression and PCa risk, a two-sample Mendelian randomization (MR) analysis was performed using the TwoSampleMR(0.6.21) package [65]. Exposure data were obtained from eQTL datasets in the IEU OpenGWAS database (ABCC4: eqtl-a-ENSG00000125257; PRKCA: eqtl-a-ENSG00000154229; ACOX2: eqtl-a-ENSG00000168306; F5: eqtl-a-ENSG00000198734). FERMT2 was excluded because no suitable instrumental SNP was available. Outcome data were obtained from FinnGen Release 12 (CD2_INSITU_PROSTATE_EXALLC). Independent instrumental variables were selected using LD clumping (r^2^ = 0.001, 10,000 kb), and weak instruments were excluded based on F-statistics >10. The inverse variance weighted (IVW) method was used as the primary MR approach, while the Wald ratio method was applied for ACOX2 because only one SNP was available. Sensitivity analyses were performed to evaluate the robustness of the estimates [66]. The MR analysis was interpreted as evidence for genetic association between predicted gene expression and PCa risk rather than evidence of a TCS-driven causal pathway.

### 4.12. Single-Cell Level Validation of PCa

The single-cell dataset (GSE193337) was first quality-controlled using the “Seurat(5.1.0)” package. Cell filtering criteria included a minimum of 200 genes per cell and ≤20% mitochondrial and ribosomal gene expression, and genes were required to be expressed in at least 3 cells for downstream analysis. Normalization was performed using the Log Normalize method, and batch effects were removed using the “harmony(1.2.3)” package. Appropriate PC values were selected according to the ElbowPlot, and cell clustering was conducted using the FindClusters feature of the Seurat package (resolution = 1.5). The results were visualized using the uniform manifold approximation and projection (UMAP) method [67]. Cell types were annotated using the SingleR(2.4.1) package based on reference transcriptomic profiles, and epithelial cells were extracted and interpreted as a tumor-enriched epithelial population for exploratory analysis based on classic epithelial marker expression. We acknowledge that malignant cell identity was not independently confirmed using inferCNV or CNV-based approaches. The distribution of selected candidate genes across cell populations was visualized using the FeaturePlot function. Furthermore, a predicted TCS-associated target gene-set score was calculated using the UCell(2.6.2) package based on a 5-gene signature (FERMT2, F5, PRKCA, ABCC4, and ACOX2), and “irGSEA(2.1.5)” was used for visualizing gene set activity with density plots [68]. This score represents the relative expression enrichment of predicted TCS-related target genes and does not indicate cellular TCS exposure levels.

### 4.13. Statistical Analysis

During data preprocessing, Strawberry Perl 5.32.1.1 was used for GEO metadata curation, platform annotation parsing, and probe-level expression matrix processing; raw microarray files (CEL/IDAT) were not used [69]. All statistical analyses were performed in the R language environment (v4.1.2). Statistical analyses were performed using appropriate tests selected based on data distribution characteristics and experimental design. Student’s t-test was employed to compare normally distributed continuous variables between two independent groups (e.g., GSVA-derived pathway activity scores), whereas the Wilcoxon rank-sum test was utilized for non-normally distributed continuous data (e.g., immune cell infiltration abundance, gene expression levels). The Wilcoxon signed-rank test was applied for comparisons of paired samples. Spearman’s rank correlation coefficient was used for correlation studies between variables. For multiple test correction, a significance threshold of *p* < 0.05 was set, and the Benjamini–Hochberg method was applied to control the false discovery rate (FDR) [70].

In Mendelian randomization (MR) analyses, instrumental variables were selected and filtered following established guidelines, including genome-wide significant association with the exposure (*p* < 5 × 10^−8^), linkage disequilibrium–based clumping to ensure independence (r^2^ = 0.001, 10,000 kb), use of the 1000 Genomes Project as the reference panel, exclusion of weak instruments (F-statistics > 10), and correction for multiple testing using the Bonferroni method [71].

## 5. Conclusions

We integrated computational MR, molecular docking/molecular dynamics, and single-cell transcriptomics to prioritize genes and pathways that may connect predicted TCS-related molecular alterations with PCa. Five candidate genes—FERMT2, F5, PRKCA, ABCC4, and ACOX2—were repeatedly highlighted across transcriptomic signatures and network analyses. Docking and MD analyses supported structural compatibility of the modeled TCS–ABCC4 and TCS–ACOX2 complexes under the simulated conditions, but do not establish physiological binding or target engagement. MR provided genetic evidence consistent with a potential association between higher ABCC4 expression and PCa risk, but it does not, on its own, establish TCS as the upstream driver. Single-cell analyses showed ABCC4 and ACOX2 enrichment in tumor-enriched epithelial populations, and pathway analyses converged on lipid metabolism and glutathione/redox processes. Together, these findings generate testable hypotheses that TCS-related molecular targets, including ABCC4-associated networks, may participate in fatty-acid oxidation and redox homeostasis relevant to PCa biology, potentially shaping inflammatory–metabolic states that may be associated with PCa progression. Future studies should evaluate target engagement and downstream functional effects under environmentally relevant exposure conditions.

## Data Availability

The original contributions presented in this study are included in the article/Supplementary Materials. Further inquiries can be directed to the corresponding author.

## Supplementary Materials

The following supporting information can be downloaded at https://www.mdpi.com/article/10.3390/ijms27209143/s1.

## Abbreviations

The following abbreviations are used in this manuscript:

ADT	Androgen-deprivation therapy
AR	Androgen receptor
CDF	Cumulative distribution function
CIBERSORT	Cell-type Identification By Estimating Relative Subsets Of RNA Transcripts
EDC	Endocrine-disrupting chemical
eQTL	Expression quantitative trait loci
FDR	False discovery rate
GEO	Gene Expression Omnibus
GLM	Generalized linear model
GO	Gene Ontology
GSVA	Gene set variation analysis
GWAS	Genome-wide association studies
IVW	Inverse variance weighting
KEGG	National Center for Biotechnology Information
MR	Mendelian randomization
NCBI	National Center for Biotechnology Information
NHANES	National Health and Nutrition Examination Survey
PCA	Principal component analysis
PCa	Prostate cancer
PDB	Protein Data Bank
PSA	Prostate-specific antigen
RF	Random forest
ROC	Receiver operating characteristic
SMR	Summary-data-based Mendelian randomization
ssGSEA	Single-sample gene set enrichment analysis
TCGA	The Cancer Genome Atlas
TCS	Triclosan
TOM	Topological overlap matrix
UMPA	Uniform manifold approximation and projection
