# Supplementary material for: Potential Molecular Links Between Triclosan Exposure and Prostate Cancer: An Integrative Computational Analysis
Source: Int J Mol Sci. 2026 Oct 15;27(20):9143. doi: 10.3390/ijms27209143 (PMC13704932; doi:10.3390/ijms27209143)
Supplement: Supplementary file 1 [file ijms-27-09143-s001.zip › Supplementary Figure S1.pdf]

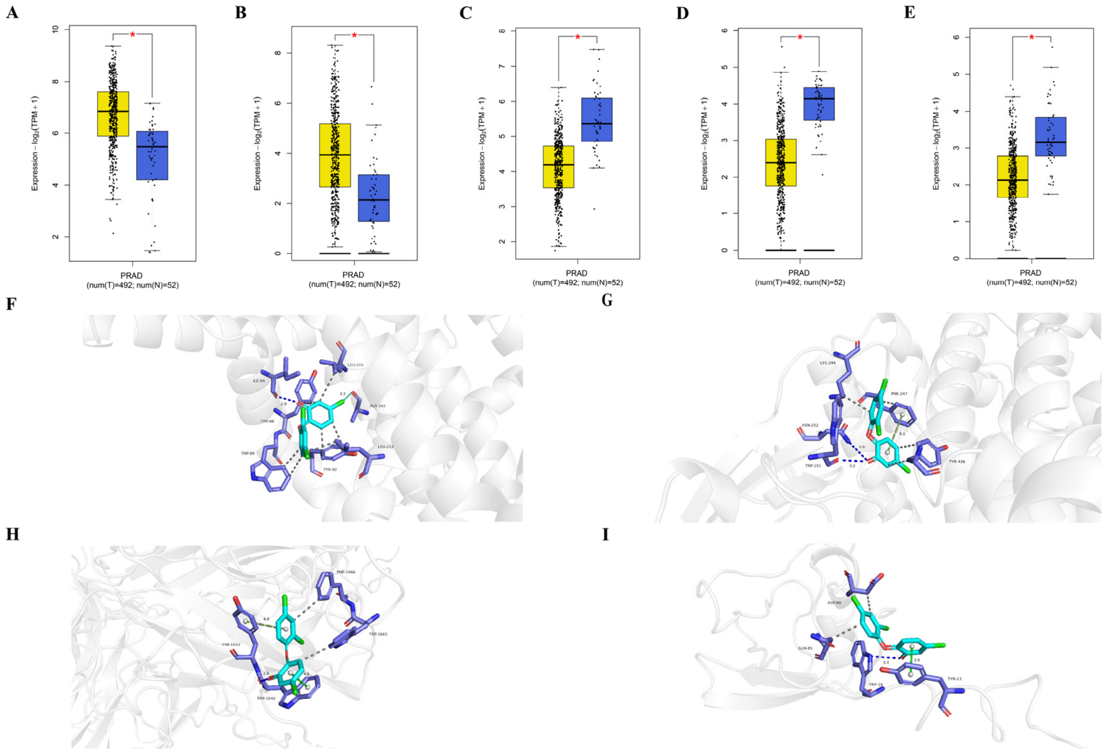

Supplementary Figure S1(A-E) TCGA validation boxplot of ABCC4, F5, FERMT2, ACOX2, and PRKCA machine learning models (F-G) Combined docking plots of TCS with ABCC4, TCS with ACOX2,TCS with F5, and TCS with FERMT2
